# Patient-Reported Racial and Ethnic Disparities in Patients With Ulcerative Colitis: Results From the National Health and Wellness Survey

**DOI:** 10.1093/crocol/otae048

**Published:** 2024-09-18

**Authors:** Sabree C Burbage, Kathryn L Krupsky, M Janelle Cambron-Mellott, Nate Way, Aarti A Patel, Julia J Liu

**Affiliations:** Population Health Research, Janssen Scientific Affairs, LLC, Horsham, PA, USA; Real-World Evidence, Cerner Enviza, an Oracle Company, Kansas City, MO, USA; Division of Gastroenterology, Morehouse School of Medicine, Atlanta, GA, USA; Real-World Evidence, Cerner Enviza, an Oracle Company, Kansas City, MO, USA; Population Health Research, Janssen Scientific Affairs, LLC, Horsham, PA, USA; Division of Gastroenterology, Morehouse School of Medicine, Atlanta, GA, USA

**Keywords:** ulcerative colitis, inflammatory bowel disease, National Health and Wellness Survey, condition severity, race/ethnicity, patient-reported outcomes

## Abstract

**Background:**

Ulcerative colitis (UC) is an inflammatory condition characterized by chronic, disabling gastrointestinal symptoms that can have detrimental effects on psychological, social, and professional quality of life. Few studies have examined patient-reported outcomes (PROs) and economic outcomes among individuals with varying UC severity and across different racial/ethnic groups.

**Methods:**

This cross-sectional study assessed sociodemographic data, PROs, and economic outcomes for participants from the National Health and Wellness Survey (2018, 2019, and 2020) with UC. Multivariable analyses were used to assess the association of self-reported UC severity and race/ethnicity with health-related quality of life (HRQoL), work productivity and activity impairment (WPAI), healthcare resource utilization (HCRU), and medical costs.

**Results:**

This study included 1500 participants with UC (1150 non-Hispanic White, 99 non-Hispanic Black, and 251 Hispanic). Moderate/severe disease was associated with significantly worse HRQoL and WPAI, greater HCRU, and higher direct medical costs than mild UC. Compared with non-Hispanic White participants, non-Hispanic Black participants reported better HRQoL, whereas Hispanic participants reported more HCRU and higher medical costs. Race/ethnicity significantly interacted with UC severity level in predicting labor force participation.

**Conclusions:**

Participants with moderate/severe disease had worse outcomes than those with mild UC. Additionally, racial/ethnic differences were found in HRQoL, employment, WPAI, HCRU, and direct medical costs. Notably, Hispanic participants showed distinct patterns, particularly in how disease severity influenced employment outcomes. Further research is needed to better understand the differential burden among patients across racial/ethnic groups.

## Graphical Abstract

**Graphical Abstract:**
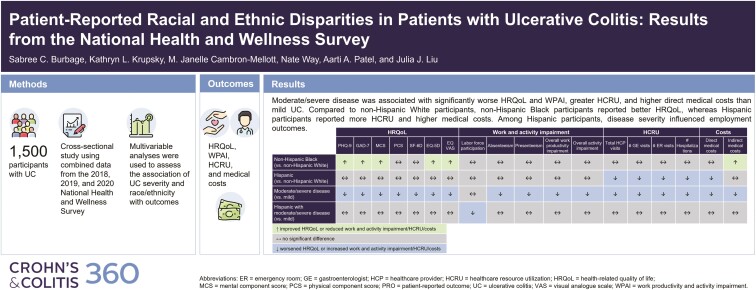

## Introduction

Ulcerative colitis (UC) is a chronic, progressive condition and 1 of the 2 more common forms of inflammatory bowel disease (IBD).^1^ Ulcerative colitis is characterized by continuous mucosal inflammation that initiates in the rectum and extends into the colon. In 2017, the global prevalence of UC was estimated to be 0.08–505 cases per 100 000 persons, and 6.8 million people were affected worldwide.^2,3^ Ulcerative colitis is most common in industrialized countries, but its incidence is quickly rising in developing countries.^2,4^ Although UC affects all age groups, it is most often diagnosed in adults 20–30 and 50–80 years of age.^5^ Symptoms of UC may include bloody diarrhea, abdominal pain, and rectal urgency, with patients experiencing variable rates of relapse and remission.^1^ These negatively impact patients’ health-related quality of life (HRQoL), such that patients with UC often experience more fatigue, social and professional impairment, low self-esteem, and psychiatric comorbidities.^6–8^ Additionally, though UC can be adequately managed with pharmacological/therapeutic treatment, approximately 10% of patients require surgery within the first year after diagnosis and up to 30% of patients require surgery in their lifetime.^9^

Ulcerative colitis is a costly condition given its chronic nature and need for multidisciplinary care teams.^10^ A 2020 study estimated the average lifetime healthcare cost of a UC patient based on outpatient visits, inpatient visits, pharmacy costs, and emergency room costs to be $405 496.^11^ Disease severity also plays a differential role in the impact of IBD burden, with patients with severe disease reporting greater healthcare resource utilization (HCRU) and higher direct medical costs than those with mild disease.^12^ Additionally, UC severity is associated with worse HRQoL and increased work productivity loss.^12^ As productivity loss due to sick leave is a major driver of indirect costs among patients with UC, patients with severe UC experience higher indirect costs than those with mild UC.^12,13^ Furthermore, considering self-reported severity is crucial for understanding disease burden, since symptoms identified as most problematic by patients may not be considered the cardinal symptoms assessed in clinical practice, and physicians often underestimate the impact of IBD symptoms on patients’ lives.^14–16^

Evidence describing how race/ethnicity influences outcomes for patients with UC is limited. Race and ethnicity are social constructs that refer to the categorization of people based on perceived shared physical traits and culture, respectively.^17^ Racial/ethnic differences in healthcare access are well documented and contribute to variations in patients’ experiences.^18^ For example, among non-White populations in the United States, downstream effects of systemic racism contribute to common obstacles to accessing healthcare, such as limited employment options, insufficient insurance coverage, and language barriers^19^; such obstacles likely contribute to poorer outcomes for patients in these groups. Indeed, previous literature shows that Black individuals have longer symptom durations and are older at the time of IBD diagnosis than non-Black individuals.^20,21^ Once diagnosed, Black patients with IBD are also less likely to receive care from IBD specialists than similar White patients.^22,23^ Moreover, the ratio of disease prevalence to hospitalization and mortality rates is disproportionately higher among Black and Hispanic patients in the United States than among White patients,^24,25^ and Black and Hispanic patients with UC are 25%–50% less likely to undergo colectomy to treat their symptoms than White populations.^10^ Notably, comprehensive reviews on how race and ethnicity influence disease severity and their implications on humanistic and economic outcomes, especially beyond the comparison of White patients to all other races/ethnicities, are limited. A better understanding of the interaction of self-reported race/ethnicity and disease severity among patients diagnosed with UC on outcomes such as HRQoL, work productivity and activity impairment (WPAI), healthcare resource utilization (HCRU), and medical costs is needed to optimize care for all patients and reduce the overall burden associated with UC. Therefore, the objective of this study was to evaluate the impact of racial/ethnic disparities and disease severity on the humanistic and economic burden associated with UC in the United States.

## Methods

### Study Design

We used data from the 2018, 2019, and 2020 US National Health and Wellness Survey (NHWS), a cross-sectional, self-administered, Internet-based survey of approximately 75 000 adults (≥18 years) annually.^26^ The survey is designed to capture the health and disease burden of the general adult population for over 165 self-reported therapeutic conditions.^27,28^ Potential participants were recruited through opt-in e-mails, co-registration with panel partners, e-newsletter campaigns, banner placements, and affiliate networks. All NHWS participants completed an in-depth demographic registration profile. A quota sampling technique was used to ensure that the demographic composition (ie, age, gender, and race/ethnicity) of the NHWS sample was representative of the adult population in the United States. The NHWS was reviewed by Pearl Institutional Review Board (IRB; Indianapolis, IN) and granted exemption status, hence a separate IRB review was not required for this study. A study protocol and analysis plan were developed a priori to guide all aspects of the study.

### Study Population

**Figure 1.**
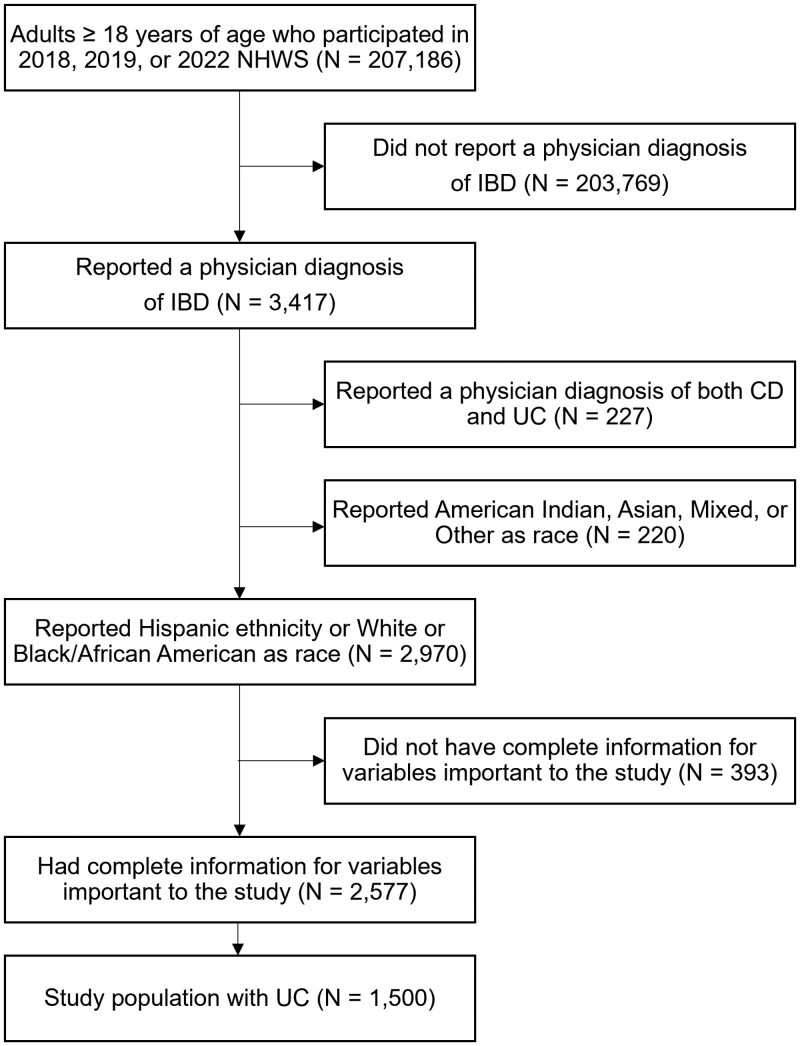
Study participant flow chart. Abbreviations: CD = Crohn’s disease; IBD = inflammatory bowel disease; NHWS = National Health and Wellness Survey; UC = ulcerative colitis.

Participants included in the analytic sample met the following inclusion criteria at the time of the study: ≥18 years old, resident of the United States, self-reported ever receiving a physician diagnosis of UC, and identified as non-Hispanic White (having origins in any of the original peoples of Europe, the Middle East, or North Africa), non-Hispanic Black/African American (having origins in any of the black racial groups of Africa), or Hispanic (of Cuban, Mexican, Puerto Rican, South or Central American, or other Spanish culture or origin, regardless of race).^17^ Additionally, participants needed to have complete data for outcomes relevant to the study (Figure 1). Participants who reported a diagnosis of Crohn’s disease or identified as a different race/ethnicity were excluded from the study.

### Sociodemographic and Clinical Characteristics

We evaluated self-reported sociodemographic (ie, sex, age, marital status, education level, employment status, household income, health insurance) and clinical characteristics (ie, UC severity, body mass index, current prescription medication use, sleep problems/symptoms, Charlson Comorbidity Index [CCI]^29^). Self-reported UC severity was captured as the participants’ rating of their UC as either mild, moderate, or severe. Due to sample size limitations, moderate and severe UC were grouped into one category to allow for the evaluation of disease severity as an effect modifier. We also assessed various health behaviors, including alcohol use, cigarette smoking, and exercise over the past 30 days, as well as previous clinical trial participation and patients’ willingness to self-monitor and regulate their health behaviors using the Patient Activation Measure (PAM).^29^ See Supplementary Materials for more details on self-reported UC severity, PAM, and clinical trial participation.

### Study Outcomes

Humanistic burden was assessed through patient-reported outcomes (PROs) of depression and anxiety, HRQoL, labor force participation, and WPAI. Depression and anxiety severity were assessed using the Patient Health Questionnaire-9 (PHQ-9) and Generalized Anxiety Disorder Assessment (GAD-7), respectively. HRQoL was assessed using the Medical Outcomes Study 36-Item Short Form Survey Instrument (SF-36v2), physical component summary (PCS) and mental component summary (MCS), the SF-6D, and the 5-level EQ-5D version (EQ-5D-5L), the last of which consists of the EQ-5D descriptive system and EQ visual analog scale (VAS).^30,31^ Higher PHQ-9 and GAD-7 scores indicate worse depressive/anxious symptomology and higher MCS, PCS, EQ-5D, SF-6D, and EQ VAS scores indicate better HRQoL. Labor force participation was derived from NHWS data through coding employment status as currently in the labor force (ie, full-time employed, part-time employed, self-employed, or not unemployed but looking for work) or not currently in the labor force (ie, retired, disabled, homemaker, student, or not employed and not looking for work). Work productivity was assessed using the WPAI questionnaire.^32^ Only participants who reported a work status of full-time, part-time, or self-employed provided data for absenteeism, presenteeism, and overall work impairment.

Economic burden was assessed through HCRU and direct and indirect costs. HCRU included the number of self-reported visits to any healthcare provider (HCP), gastroenterologist (GE), and emergency room (ER), and hospitalizations over the past 6 months. Direct medical costs were imputed using data from the region-specific Medical Expenditure Panel Survey and included costs of an average HCP visit, ER visit, and hospitalization based on the sum of direct payments of care provided during the year, including out-of-pocket payments and payments by private insurance, Medicaid, Medicare, and other sources.^33^ Indirect costs were those associated with work productivity impairment and were calculated using estimated wages/salaries for each participant with data from the US Bureau of Labor Statistics. This cost analysis approach has been used in prior research in the NHWS.^34,35^ Detailed descriptions of the study outcomes and how they were calculated are included in the Supplementary Materials.

### Data Analysis

Descriptive statistics were used to characterize the UC cohort; results were reported as frequencies or percentages for categorical variables and means with standard deviations (SDs) for continuous variables. Bivariate analyses were used to examine sociodemographic and clinical characteristics across UC severity groups and race/ethnicity and inform covariate selection for multivariable analyses (see Supplementary Tables 1 and 3).

To assess associations between UC severity, race/ethnicity, and outcomes of interest, multivariable analyses were conducted by constructing generalized linear regression models (ie, linear model for SF-36 and EQ-5D, binary logistic model for labor force participation, and negative binomial model for all other outcomes). The same covariates were included in each regression model for all outcomes, enabling comparisons of the magnitude of the association for each independent variable. Models controlled for age, gender, marital status, educational attainment, household income, health insurance coverage, weight status, smoking status, alcohol use, and CCI score. Adjusted means, mean differences (MD), and *P* values were reported.

Separate covariate-adjusted models were used to assess whether the relationship between UC severity and specific outcomes depended on race/ethnicity. The heterogeneity of the association was determined using an interaction term between race/ethnicity and UC severity, which was added to each covariate-adjusted model. The statistical significance of the interaction term was assessed using type III analyses (the *F* test for linear models and the likelihood ratio test [LRT] for logistic or negative binomial models). For models where the interaction term was statistically significant, interactions were probed via stratification, and covariate-adjusted estimates were interpreted in the context of the interaction term. All analyses were conducted using SAS 9.4.

## Results

### Sociodemographic Characteristics and Study Population

A total of 1500 participants who self-reported receiving a physician diagnosis of UC were included in this study. Of these participants, 76.7% identified as non-Hispanic White, 16.7% as Hispanic, and 6.6% as non-Hispanic Black/African American (Table 1). There was a lower proportion of female participants in the non-Hispanic Black group than in the non-Hispanic White group (47.5% vs. 60.5%; *P* = .036). Mean age was lower among non-Hispanic Black (38.48 [SD: 16.08]) and Hispanic (38.45 [SD: 14.27]) groups than among non-Hispanic White participants (52.48 [SD: 16.28]; both *P* < .001). More Hispanic participants reported moderate and severe UC than non-Hispanic White participants (moderate: 37.1% vs. 26.1%, *P *= . 002; severe: 9.6% vs. 5.3%, *P* = .035). There were no differences in CCI scores or physician-diagnosed comorbidities by race/ethnicity, except that more non-Hispanic White participants reported physician-diagnosed mental health conditions than non-Hispanic Black participants (38.8% vs. 21.2%, *P* = .002). A greater proportion of Hispanic participants were employed full time than non-Hispanic White participants (52.6% vs. 36.9%; *P* < .001); however, the distribution of household income was comparable across race/ethnicity groups. A greater proportion of Hispanic participants reported being uninsured and having commercial health insurance than non-Hispanic White participants (uninsured: 14.7% vs. 7.7%, *P* = .001; commercially insured: 59.4% vs 50.7%, *P* = .039). A greater proportion of non-Hispanic White participants reported being on Medicare than non-Hispanic Black (31.5% vs. 16.2%; *P* = .006) and Hispanic (10.8%; *P* < .001) participants (Table 1). This is consistent with a greater proportion of non-Hispanic White participants being retired than non-Hispanic Black (27.9% vs. 9.1%; *P* < .001) and Hispanic (11.6%; *P* < .001) participants.

**Table 1.** Sociodemographic and clinical characteristics of participants with self-reported ulcerative colitis.

	Non-Hispanic White (*n* = 1150)	Non-Hispanic Black (*n* = 99)	Hispanic (*n* = 251)
Female, *n* (%)	696 (60.5%)	47 (47.5%)^*^	138 (55.0%)
Age in years, mean (SD)	52.48 (16.28)	38.48 (16.08)^*^	38.45 (14.27)^†^
Marital status			
Married/living with partner	718 (62.4%)	29 (29.3%)^*^	125 (49.8%)^†^^‡^
Single, not married/divorced/separated/widowed	429 (37.3%)	70 (70.7%)^*^	124 (49.4%)^†^^‡^
Decline to answer	3 (0.3%)	0 (0.0%)	2 (0.8%)
Education, *n* (%)			
Less than high school	7 (0.6%)	2 (2.0%)	6 (2.4%)^†^
Completed some high school	21 (1.8%)	8 (8.1%)^*^	16 (6.4%)^†^
High school graduate or equivalent (eg, GED)	154 (13.4%)	12 (12.1%)	31 (12.4%)
Completed some college, but no degree	238 (20.7%)	20 (20.2%)	51 (20.3%)
Associate degree	144 (12.5%)	17 (17.2%)	29 (11.6%)
College graduate	280 (24.3%)	18 (18.2%)	79 (31.5%)^‡^
Completed some graduate school, but no degree	57 (5.0%)	5 (5.1%)	11 (4.4%)
Completed graduate school	245 (21.3%)	16 (16.2%)	28 (11.2%)^†^
Decline to answer	4 (0.3%)	1 (1.0%)	0 (0.0%)
Employment status, *n* (%)			
Employed full time	424 (36.9%)	44 (44.4%)	132 (52.6%)^†^
Self-employed	78 (6.8%)	9 (9.1%)	15 (6.0%)
Employed part time	85 (7.4%)	17 (17.2%)^*^	30 (12.0%)
Homemaker	73 (6.3%)	3 (3.0%)	19 (7.6%)
Retired	321 (27.9%)	9 (9.1%)^*^	29 (11.6%)^†^
Student	16 (1.4%)	8 (8.1%)^*^	8 (3.2%)
Long-term disability	84 (7.3%)	2 (2.0%)	12 (4.8%)
Not employed (whether looking for work or not)	69 (6.0%)	7 (7.1%)	6 (2.4%)
Household income, *n* (%)			
<$25 000	166 (14.4%)	22 (22.2%)	39 (15.5%)
$25 000 to <$50 000	235 (20.4%)	25 (25.3%)	53 (21.1%)
$50 000 to <$100 000	409 (35.6%)	29 (29.3%)	74 (29.5%)
$100 000+	299 (26.0%)	23 (23.2%)	77 (30.7%)
Decline to answer	41 (3.6%)	0 (0.0%)	8 (3.2%)
Health insurance, *n* (%)			
Not insured	88 (7.7%)	11 (11.1%)	37 (14.7%)^†^
Commercially insured	583 (50.7%)	54 (54.5%)	149 (59.4%)^†^
Medicaid	85 (7.4%)	13 (13.1%)	21 (8.4%)
Medicare	362 (31.5%)	16 (16.2%)^*^	27 (10.8%)^†^
Other type of insurance/unsure	32 (2.8%)	5 (5.1%)	17 (6.8%)^†^
PAM score^a^, mean (SD) PAM level^b^, *n* (%)	62.28 (11.44)	58.41 (14.25)^*^	58.03 (13.55)^†^
Level 1	87 (7.6)	25 (25.3)^*^	56 (22.3)^†^
Level 2	202 (17.6)	19 (19.2)	45 (17.9)
Level 3	656 (57.0)	39 (39.4)^*^	114 (45.4)^†^
Level 4	205 (17.8)	16 (16.2)	36 (14.3)
Severity of condition^c^, *n* (%)			
Mild	789 (68.6%)	61 (61.6%)	134 (53.4%)^†^
Moderate	300 (26.1%)	27 (27.3%)	93 (37.1%)^†^
Severe	61 (5.3%)	11 (11.1%)	24 (9.6%)^†^
CCI score, *n* (%)			
0	623 (54.2%)	55 (55.6%)	130 (51.8%)
1	251 (21.8%)	19 (19.2%)	48 (19.1%)
2	132 (11.5%)	11 (11.1%)	34 (13.5%)
3	144 (12.5%)	14 (14.1%)	39 (15.5%)
Comorbidities (physician-diagnosed), *n* (%)			
Skin condition	429 (37.3%)	27 (27.3%)	81 (32.3%)
Liver condition	75 (6.5%)	7 (7.1%)	23 (9.2%)
Eye condition	393 (34.2%)	37 (37.4%)	73 (29.1%)
Joint condition	443 (38.5%)	39 (39.4%)	87 (34.7%)
Mental health condition	446 (38.8%)	21 (21.2%)^*^	78 (31.1%)
Urological condition	246 (21.4%)	16 (16.2%)	52 (20.7%)
Sleep conditions	382 (33.2%)	29 (29.3%)	65 (25.9%)
Ever participated in a clinical trial, *n* (%)	191 (16.6%)	39 (39.4%)^*^	97 (38.6%)^†^

Sociodemographic characteristics were assessed using bivariate analyses.

^a^PAM scores range from 0 to 100 where higher scores indicate higher levels of activation.

^b^PAM scores correlate to one of four levels of patient activation: Level 1 = disengaged and overwhelmed; Level 2 = becoming aware but still struggling; Level 3 = taking action; Level 4 = maintaining behavior and pushing further.

^c^Due to sample size limitations, moderate and severe CD were grouped into one category to allow for the evaluation of disease severity as an effect modifier.

^d^This refers to all clinical trials, not just IBD-related trials.

^*^ *α *< 0.05 between non-Hispanic Black and non-Hispanic White participants.

^†^ *α* < 0.05 between Hispanic and non-Hispanic White participants.

^‡^ *α* < 0.05 between Hispanic and non-Hispanic Black participants. Results were adjusted for multiple comparisons with the Bonferroni correction.

Abbreviations: CCI = Charlson Comorbidity Index; PAM = patient activation measure; SD = standard deviation.

Patient activation scores were, on average, lower among non-Hispanic Black (58.41 [SD: 14.25]) and Hispanic (58.03 [SD: 13.55]) participants than non-Hispanic White participants (62.28 [SD: 11.44]; *P* = .007 and *P* < .001, respectively). Notably, the study population was highly engaged, with 71.1% of participants reporting a PAM level of 3 (ie, taking action and gaining control) or 4 (ie, maintaining behaviors and pushing further). Previous participation in any clinical trial was significantly higher among non-Hispanic Black (39.4%) and Hispanic participants (38.6%) than among non-Hispanic White participants (16.6%; both *P* < .001).

### Study Outcomes by Disease Severity—Main Effects

**Figure 2.**
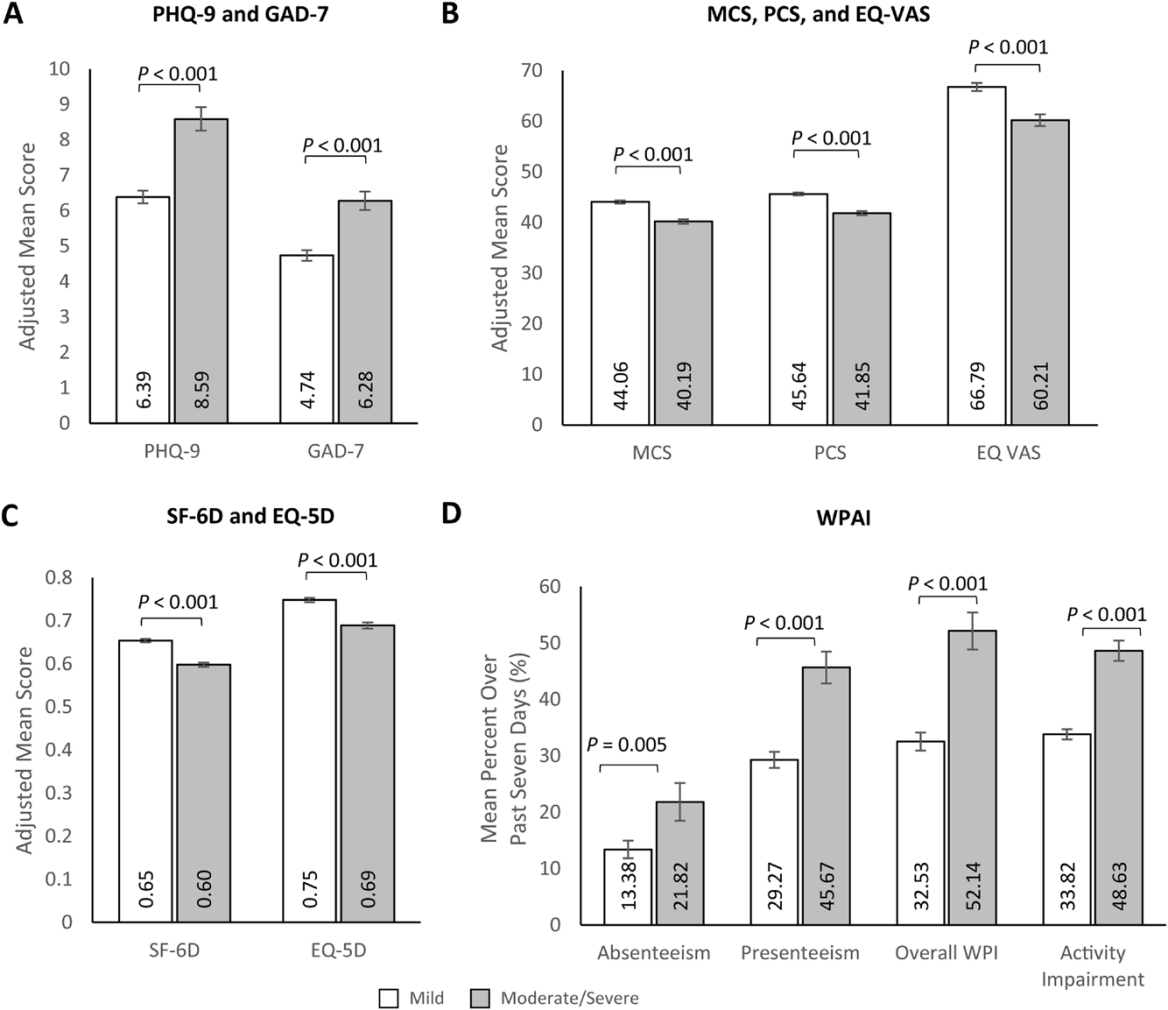
Multivariable analyses of patient-reported outcomes of (A) PHQ-9 and GAD-7, (B) MCS, PCS, and EQ VAS, (C) SF-6D and EQ 5D, and (D) WPAI by ulcerative colitis severity. Note: Lower PHQ-9 and GAD-7 scores and higher MCS, PCS, EQ VAS, SF-6D, and EQ-5D scores indicate better HRQoL. Error bars represent standard error. Presenteeism was only assessed among participants who worked >0 hours over the last 7 days. PHQ-9, GAD-7, and WPAI outcomes were modeled using a log link with a negative binomial distribution; exp(β) = rate ratio. MCS, PCS, EQ VAS, SF-6D, and EQ-5D were modeled using an identity link with a normal distribution; β = estimated mean difference. Models control for age (continuous; set to mean = 45.19 years), gender (male [ref]; female), marital status (single/never married/decline to answer; married/living with a partner [ref]), educational attainment (less than a college degree/declined to answer; college graduate or higher [ref]), household income (<$25 000; $25 000 to <$50 000; $50 000 to <$100 000; $100 000+ [ref]), health insurance coverage (Medicare; Medicaid/VA/CHAMPUS; uninsured; commercial/TRICARE/don’t know [ref]), weight status (obese; overweight; underweight/normal weight/ declined to answer [ref]), smoking status (current smoker; former smoker; never smoker [ref]), alcohol use (drinks alcohol; does not drink alcohol [ref]), and Charlson Comorbidity Index Score (continuous; set to mean = 1.14). Abbreviations: EQ-5D-5L = EuroQol 5-Dimension 5-Level; GAD-7 = Generalized Anxiety Disorder—7 item; MCS = Mental Component Summary; PCS = Physical Component Summary; PHQ-9 = Patient Health Questionnaire-9 item; SF-6D = Short Form-6 Dimension; WPAI = Work Productivity and Activity Impairment; VAS = Visual Analog Scale.

Multivariable analyses adjusting for potential confounders showed that participants with moderate/severe UC had significantly worse self-reported depression and anxiety than those with mild UC (PHQ-9 [depression] score: 8.59 [standard error {SE}: 0.33] vs. 6.39 [SE: 0.18]; *P* < .001; GAD-7 [anxiety] score: 6.28 [SE: 0.26] vs. 4.74 [SE: 0.15]; *P* < .001; Figure 2A, Supplementary Table 1). Consistent with these outcomes, participants with moderate/severe UC had significantly worse SF-36 MCS and PCS HRQoL scores than those with mild UC (MCS: 40.19 [SE: 0.45] vs. 44.06 [SE: 0.32]; MD: −3.88 *P* < .001 and PCS: 41.85 [SE: 0.39] vs. 45.64 [SE: 0.28]; MD: −3.78; *P* < .001; Figure 2B, Supplementary Table 1). Participants with moderate/severe UC also reported worse utility scores than participants with mild UC, with an MD of −0.06 for SF-6D (0.60 [SE: 0.01] vs. 0.65 [SE: 0.01]; *P* < .001), −0.06 for EQ-5D-5L (0.69 [SE: 0.01] vs. 0.75 [SE: 0.01]; *P* < .001), and −6.58 for EQ VAS (60.21 [SE: 1.12] vs. 66.79 [0.80]; *P* < .001) scores (Figure 2C, Supplementary Table 1).

**Figure 3.**
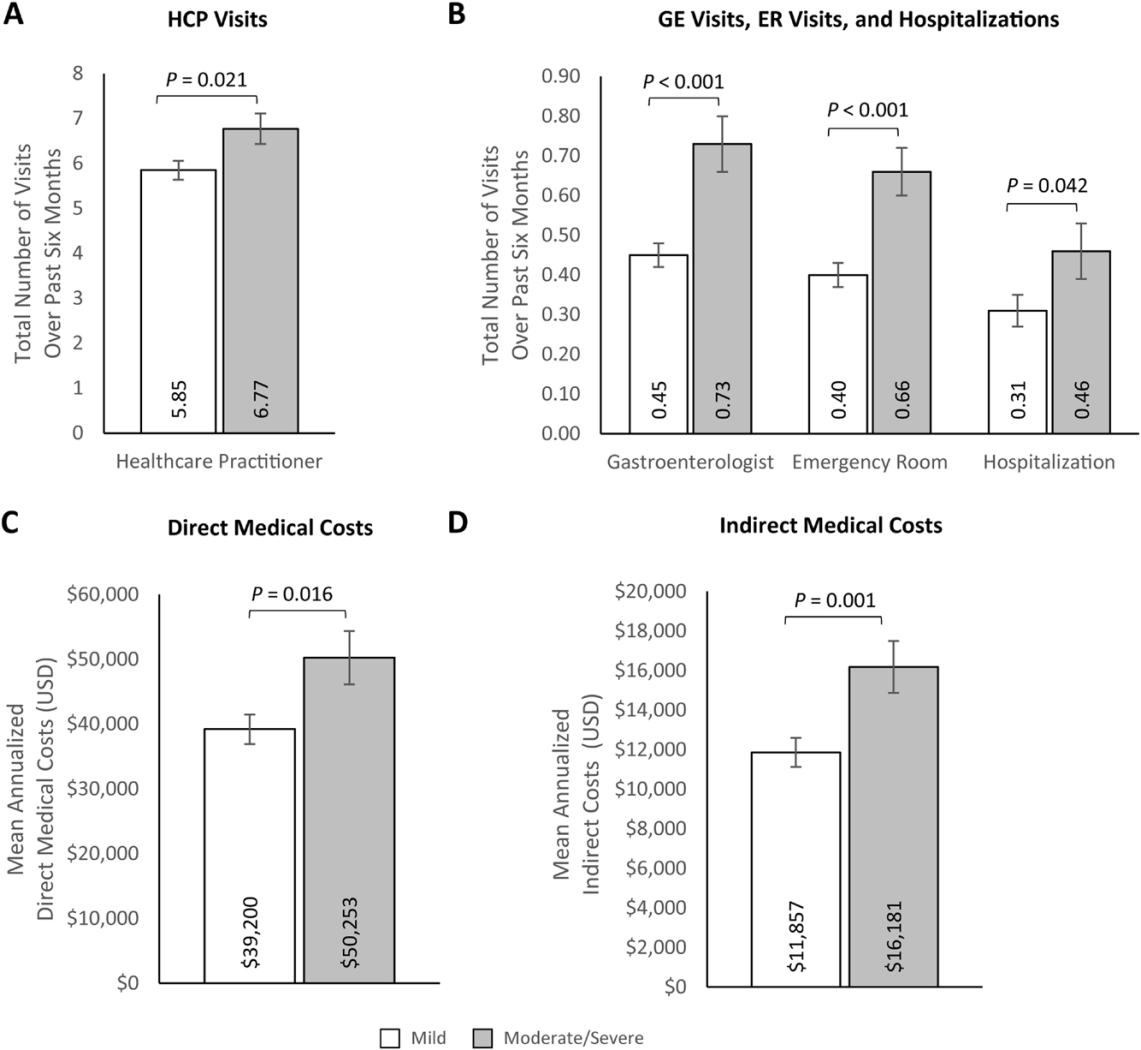
Multivariable analyses of (A) HCP visits, (B) ER visits and hospitalizations, (C) annualized direct medical costs, and (D) annualized indirect medical costs by ulcerative colitis severity. Error bars represent standard error. Indirect costs were only calculated for participants in the labor force at the time of the survey and who had a valid response (ie, non-missing) for the number of hours worked over the past seven days and the number of hours missed in the past seven days (mild *n* = 425; moderate/severe *n* = 274). Outcomes were modeled using a log link with a negative binomial distribution; exp(β) = rate ratio. Models control for age (continuous; set to mean = 45.19 years), gender (male [ref]; female), marital status (single/never married/decline to answer; married/living with a partner [ref]), educational attainment (less than a college degree/declined to answer; college graduate or higher [ref]), household income (<$25 000; $25 000 to <$50 000; $50 000 to <$100 000; $100 000+ [ref]), health insurance coverage (Medicare; Medicaid/VA/CHAMPUS; uninsured; commercial/TRICARE/don’t know [ref]), weight status (obese; overweight; underweight/normal weight/ declined to answer [ref]), smoking status (current smoker; former smoker; never smoker [ref]), alcohol use (drinks alcohol; does not drink alcohol [ref]), and Charlson Comorbidity Index Score (continuous; set to mean = 1.14). Abbreviations: ER = emergency room; HCP = healthcare practitioner; USD = United States dollars.

Participants with moderate/severe UC reported greater impairment across all WPAI measures for the past seven days than participants with mild UC (absenteeism: 21.82% [SE: 3.35%] vs. 13.38% [SE: 1.55]; *P* = .005; presenteeism: 45.67% [SE: 2.82%] vs. 29.27% [1.45%]; *P* < .001; work productivity impairment: 52.14% [SE: 3.29%] vs. 32.53% [SE: 1.63%]; *P* < .001; overall activity impairment: 48.63% [SE: 1.78%] vs. 33.82% [SE: 0.89%]; *P* < .001; Figure 2D, Supplementary Table 1). Additionally, participants with moderate/severe UC reported significantly higher HCRU than those with mild UC (HCP visits: 6.77 [SE: 0.34] vs. 5.85 [SE: 0.21]; *P* = .021; GE visits: 0.73 [SE: 0.07] vs. 0.45 [SE: 0.03]; *P* < .001; ER visits: 0.66 [SE: 0.06] vs. 0.40 [SE: 0.03]; *P* < .001; hospitalizations: 0.46 [SE: 0.07] vs. 0.31 [SE: 0.04]; *P* = .042) over the past 6 months (Figure 3A and B, Supplementary Table 1). Consistent with these results, direct medical costs were significantly higher among participants with moderate/severe UC than participants with mild UC ($50 252.95 [SE: $4130.76] vs. $39 200.33 [SE: $2292.87]; *P* = .016; Figure 3C, Supplementary Table 1). Indirect costs were also higher among participants with moderate/severe UC than among participants with mild UC ($16 181.29 [SE: $1307.74] vs. $11 856.60 [SE: $736.17]; *P* = .001; Figure 3D, Supplementary Table 1). Multivariable results for outcomes by disease severity largely aligned with previous bivariate analyses (Supplementary Table 2).

### Study Outcomes by Race/Ethnicity—Main Effects

**Figure 4.**
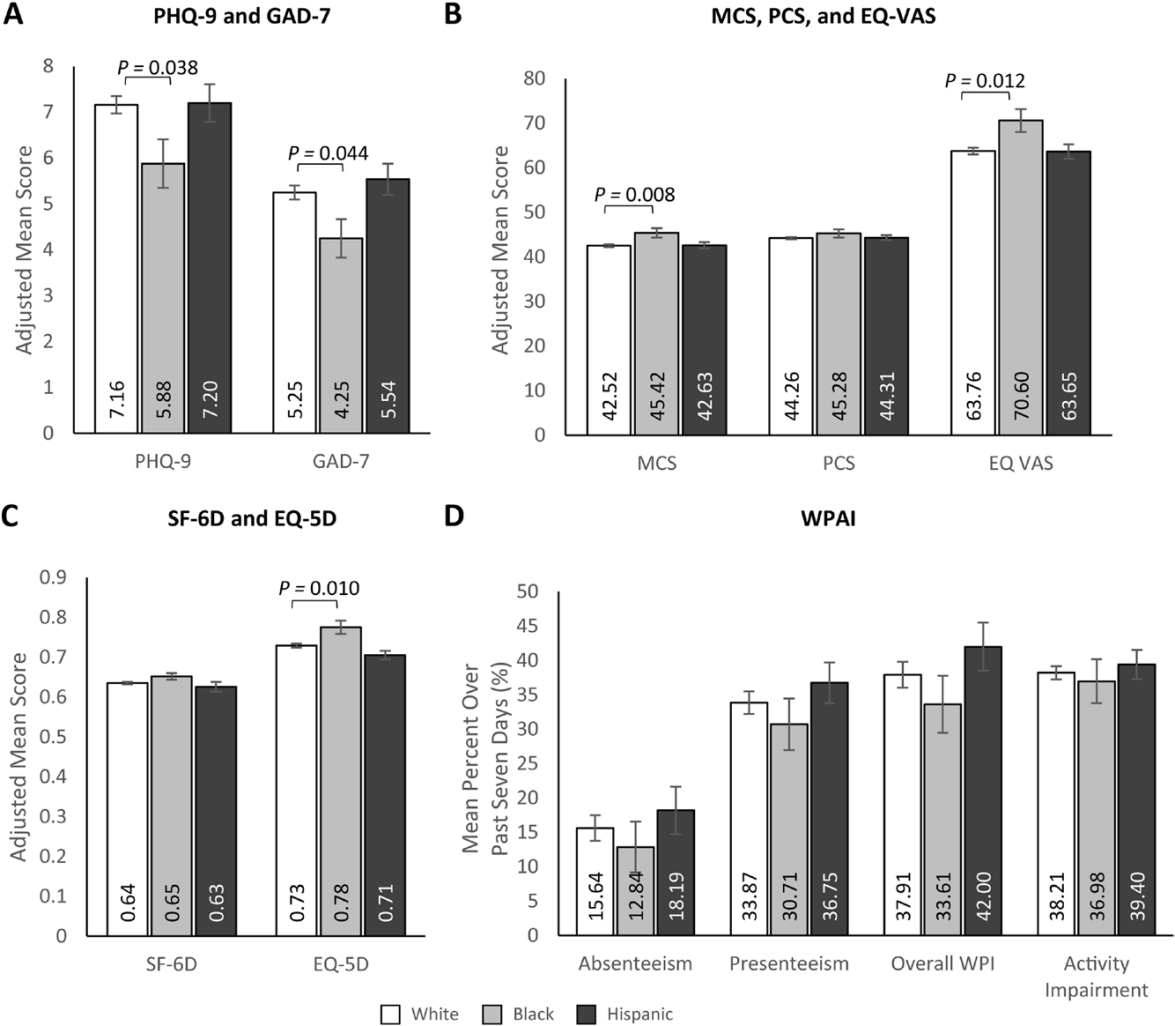
Multivariable analyses of patient-reported outcomes of (A) PHQ-9 and GAD-7, (B) MCS, PCS, and EQ VAS, (C) SF-6D and EQ 5D, and (D) WPAI by race/ethnicity. Note: Lower PHQ-9 and GAD-7 scores and higher MCS, PCS, EQ VAS, SF-6D, and EQ-5D scores indicate better HRQoL. Error bars represent standard error. Presenteeism was only assessed among participants who worked > 0 hours over the last 7 days. PHQ-9, GAD-7, and WPAI outcomes were modeled using a log link with a negative binomial distribution; exp(β) = rate ratio. MCS, PCS, EQ VAS, SF-6D, and EQ-5D were modeled using an identity link with a normal distribution; β = estimated mean difference. Models control for age (continuous; set to mean = 45.19 years), gender (male [ref]; female), marital status (single/never married/decline to answer; married/living with a partner [ref]), educational attainment (less than a college degree/declined to answer; college graduate or higher [ref]), household income (<$25 000; $25 000 to <$50 000; $50 000 to <$100 000; $100 000+ [ref]), health insurance coverage (Medicare; Medicaid/VA/CHAMPUS; uninsured; commercial/TRICARE/don’t know [ref]), weight status (obese; overweight; underweight/normal weight/ declined to answer [ref]), smoking status (current smoker; former smoker; never smoker [ref]), alcohol use (drinks alcohol; does not drink alcohol [ref]), and Charlson Comorbidity Index Score (continuous; set to mean = 1.14). Abbreviations: EQ-5D-5L = EuroQol 5-Dimension 5-Level; GAD-7 = Generalized Anxiety Disorder-7 item; MCS = Mental Component Summary; PCS = Physical Component Summary; PHQ-9 = Patient Health Questionnaire-9 item; SF-6D = Short Form-6 Dimension; WPAI = Work Productivity and Activity Impairment; VAS = Visual Analog Scale.

In multivariable analyses adjusting for potential confounders, non-Hispanic Black participants with UC had less depressive and anxious symptomology than non-Hispanic White participants (PHQ-9 [depression] score: 5.88 [SE: 0.53] vs. 7.16 [SE: 0.19]; *P* = 0.038; GAD-7 [anxiety] score: 4.25 [SE: 0.42] vs. 5.25 [SE: 0.15]; *P* = .044) (Figure 4A, Supplementary Table 3). Consistent with these findings, non-Hispanic Black participants reported better HRQoL than non-Hispanic White participants in SF-36 MCS (45.42 [SE: 1.04] vs. 42.52 [SE: 0.30]; MD: 2.91; *P* = .008), EQ-5D-5L (0.78 [SE: 0.017] vs. 0.73 [SE: 0.005]; MD: 0.05; *P *= .010), and EQ VAS (70.60 [SE: 2.58] vs. 63.76 [SE:0.75]; MD: 6.84; *P* = .012) scores, with no significant differences between Hispanic and non-Hispanic White participants (Figure 4B and C, Supplementary Table 3).

**Figure 5.**
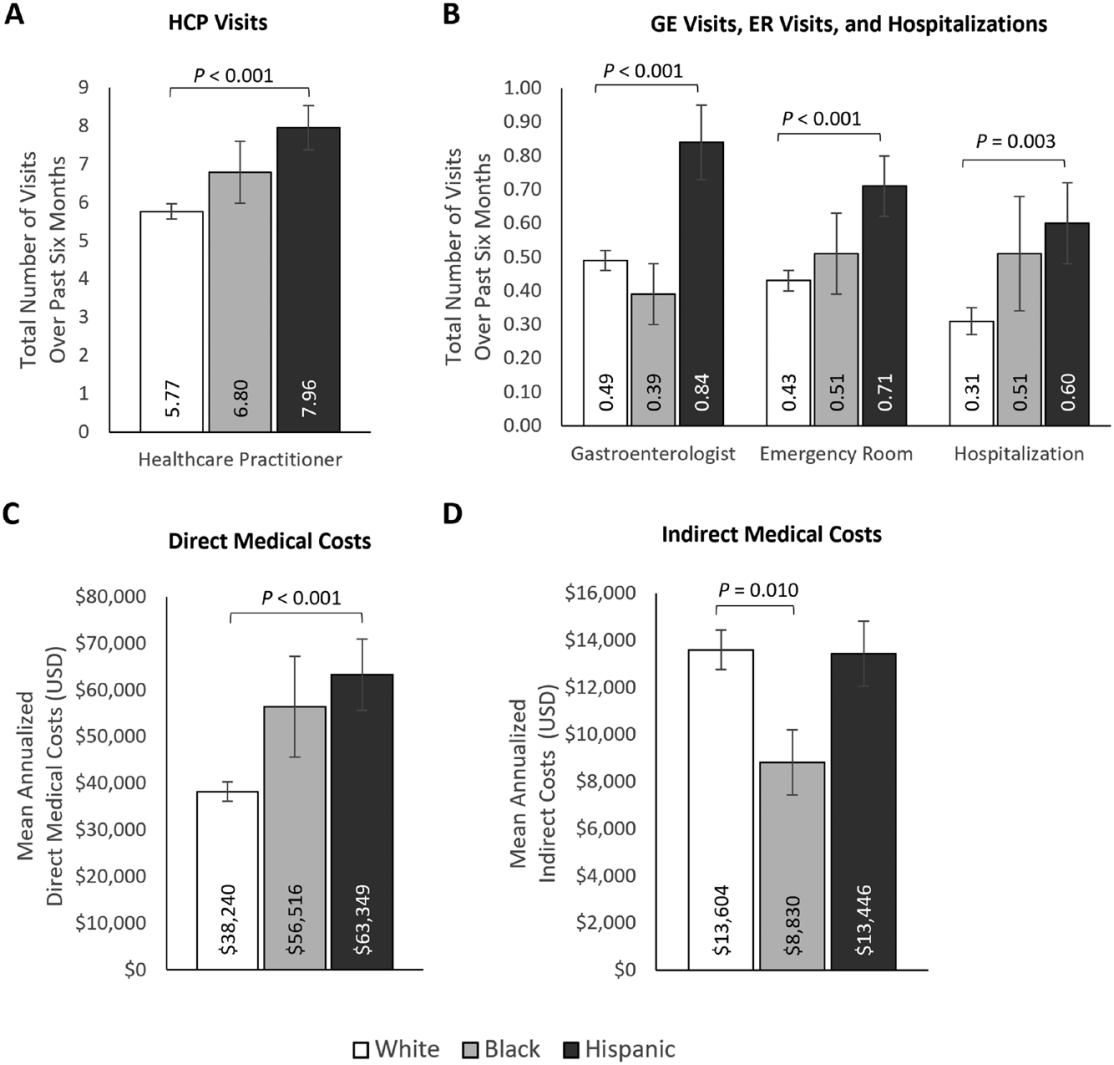
Multivariable analyses of (A) HCP visits, (B) ER visits and hospitalizations, (C) annualized direct medical costs, and (D) annualized indirect medical costs by race/ethnicity. Note: Error bars represent standard error. Indirect costs were only calculated for participants in the labor force at the time of the survey and who had a valid response (ie, non-missing) for the number of hours worked over the past seven days and the number of hours missed in the past seven days (non-Hispanic White *n* = 501; non-Hispanic Black *n *= 79; Hispanic *n* = 119). Outcomes were modeled using a log link with a negative binomial distribution; exp(β) = rate ratio. Models control for age (continuous; set to mean = 45.19 years), gender (male [ref]; female), marital status (single/never married/decline to answer; married/living with a partner [ref]), educational attainment (less than a college degree/declined to answer; college graduate or higher [ref]), household income (<$25 000; $25 000 to <$50 000; $50 000 to <$100 000; $100 000+ [ref]), health insurance coverage (Medicare; Medicaid/VA/CHAMPUS; uninsured; commercial/TRICARE/don’t know [ref]), weight status (obese; overweight; underweight/normal weight/ declined to answer [ref]), smoking status (current smoker; former smoker; never smoker [ref]), alcohol use (drinks alcohol; does not drink alcohol [ref]), and Charlson Comorbidity Index Score (continuous; set to mean = 1.14). Abbreviations: ER = emergency room; HCP = healthcare practitioner; USD = United States dollars.

Hispanic participants with UC reported significantly higher HCRU than non-Hispanic White participants, with more HCP visits (7.96 [SE: 0.58] vs. 5.77 [SE: 0.20]; *P* < .001), GE visits (0.84 [SE: 0.11] vs. 0.49 [SE: 0.03]; *P *< .001), ER visits (0.71 [SE: 0.09] vs. 0.43 [SE: 0.03]; *P* < .001) and hospitalizations (0.60 [SE: 0.12] vs. 0.31 [SE: 0.04]; *P* = .003) over the past 6 months (Figure 5A and B, Supplementary Table 3). HCRU outcomes were numerically higher among non-Hispanic Black participants compared with non-Hispanic White participants, though the differences were not statistically significant. Hispanic participants with UC reported significantly higher annualized direct medical costs than non-Hispanic White participants ($63 349 [SE: $7663] vs. $38 240 [SE: $2084]; *P *< .001; Figure 5C, Supplementary Table 3). Annualized direct medical costs were also higher among non-Hispanic Black participants than among non-Hispanic White participants but did not reach statistical significance ($56 516 [SE: $10 813]). There were no significant differences in WPAI outcomes across race/ethnicity groups (Figure 4D, Supplementary Table 3). Non-Hispanic Black participants reported lower indirect costs than non-Hispanic White participants ($8830 [SE: $1381] vs. $13 604 [SE: $839]; *P *= .010; Figure 5D, Supplementary Table 3). Bivariate analyses by race/ethnicity are presented in Supplementary Table 4.

### Interaction Between Race/Ethnicity and Disease Severity

**Figure 6.**
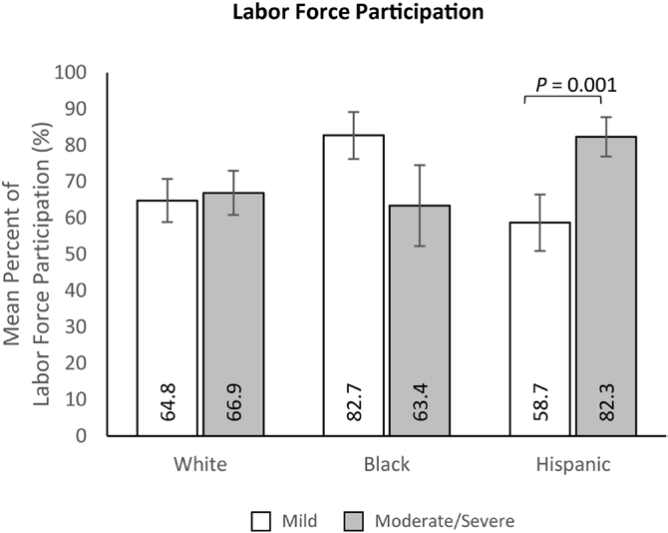
Multivariable analyses of labor force participation by race/ethnicity and condition severity. Error bars represent standard error. Results were modeled using the Likelihood ratio test. Models control for age (continuous; set to mean = 45.19 years), gender (male [ref]; female), marital status (single/never married/decline to answer; married/living with a partner [ref]), educational attainment (less than a college degree/declined to answer; college graduate or higher [ref]), household income (<$25 000; $25 000 to <$50 000; $50 000 to <$100 000; $100 000+ [ref]), health insurance coverage (Medicare; Medicaid/VA/CHAMPUS; uninsured; commercial/TRICARE/don’t know [ref]), weight status (obese; overweight; underweight/normal weight/ declined to answer [ref]), smoking status (current smoker; former smoker; never smoker [ref]), alcohol use (drinks alcohol; does not drink alcohol [ref]), and Charlson Comorbidity Index Score (continuous; set to mean = 1.14).

Multivariable analyses investigating the interaction of UC severity and race/ethnicity on outcomes showed that race/ethnicity moderated the relationship between UC severity and labor force participation (LRT *X*^2^_(2)_ = 13.88; *P* = .001). No other significant interactions were identified. Among Hispanic participants, the odds of participating in the labor force among Hispanic participants with moderate/severe UC were 3.26 times that of Hispanic participants with mild UC (82.3% [SE: 5.4%] vs. 58.7% [7.8%]; *P* = .001; Figure 6). The opposite trend was found among non-Hispanic Black participants with moderate/severe or mild UC, though the association was not statistically significant (63.4% [SE: 11.1%] vs. 82.7% [SE: 6.5%]; *P* > .05). Labor force participation was roughly equal among non-Hispanic White participants with moderate/severe UC and mild UC (66.9% [SE: 6.1%] vs. 64.8% [SE: 5.9%]; *P* > .05).

## Discussion

This study aimed to assess the humanistic and economic burden of UC relative to condition severity and race/ethnicity among 1500 patients from the United States. Results showed that moderate/severe UC was associated with significantly worse outcomes than mild UC. Non-Hispanic Black participants had less depressive and anxious symptomology, better overall HRQoL, and lower indirect costs than non-Hispanic White participants. Moreover, Hispanic participants reported higher HCRU and annualized direct medical costs than non-Hispanic White participants. We also identified a significant interaction between UC severity and race/ethnicity for labor force participation, with Hispanic participants with moderate/severe UC having higher participation in the labor force than those with mild UC, a finding not seen in non-Hispanic Black or non-Hispanic White participants.

The results of this study further validate previous studies showing the association between greater UC severity and worse outcomes, demonstrating that moderate-to-severe UC drastically affects patients’ ability to participate in activities and the workforce, increases the need for healthcare resources and direct and indirect costs.^11,12^ Participants with moderate/severe UC reported greater humanistic burden than those with mild UC, with significantly worse HRQoL scores and a significantly greater work and activity impairment. Importantly, the mean difference in HRQoL measures (MCS, PCS, SF-6D, and EQ-5D-5L) between participants with moderate/severe UC and those with mild UC exceeded clinical significance or established minimal clinically important differences,^36,37^ highlighting the profound impact of UC progression on patient quality of life. Participants with moderate/severe UC also had a greater economic burden than those with mild UC, such that they reported significantly higher HCRU and accumulated greater annual direct medical costs and indirect costs. Our study and others continue to highlight the burden among UC patients with moderate/severe disease, suggesting an undertreated and underserved patient population with substantial unmet needs.

In this study, non-Hispanic Black participants reported a lower humanistic burden than non-Hispanic White participants as indicated by lower PHQ-9 and GAD-7 scores and higher MCS, EQ-5D, and EQ VAS scores. Both MCS and PCS scores among participants in this study were lower than the national average (ie, 50), highlighting the substantial humanistic burden of UC across all race/ethnicity groups.^37^ While mean MCS scores for non-Hispanic White and Hispanic participants slightly exceeded the threshold considered indicative of clinical depression, the observed difference in MCS scores between non-Hispanic Black and non-Hispanic White participants, although statistically significant, may not meet the widely accepted MCID of 3.0.^37^ This suggests that the observed difference, while statistically notable, might not be as clinically meaningful within the context of UC. However, it is important to highlight that the other PROs in this study still suggest non-Hispanic Black participants have less depressive and anxious symptomology and better HRQoL than their non-Hispanic White counterparts. Consistent with these results, national studies report a lower lifetime risk of psychiatric disorders among non-Hispanic Black individuals than among non-Hispanic White individuals, despite experiencing higher levels of social and financial adversity.^38,39^ The “Black-White depression paradox,” which describes this phenomenon, remains a topic of ongoing research.^40,41^ One hypothesis suggests that racial socialization, the process by which individuals develop an understanding of their racial identity and learn how to navigate challenges related to race, may play a role in mitigating the impact of stressors on non-Hispanic Black individuals’ mental health.^42^ Alternatively, robust support systems within non-Hispanic Black communities may contribute to this resilience, aligning with research linking social support to reduced mental disorder prevalence.^40,43^ Racial discrepancies in response to mental health diagnostic instruments or in the extent to which mental health issues are stigmatized within race groups could also impact reported rates of mental health issues.^44,45^ Furthermore, the exclusion of homeless, institutionalized, or incarcerated individuals from epidemiologic studies due to sampling bias could mask accurate prevalence rates, given these populations often exhibit elevated rates of mental illness and are a disproportionate representation of non-Hispanic White individuals.^40^ Few studies have compared HRQoL among non-Hispanic Black and non-Hispanic White participants with UC, and subsequent research is needed to further contextualize these findings.

While many of the challenges affecting non-Hispanic Black participants described earlier may apply to Hispanic participants,^18^ our study revealed distinct patterns. In contrast to participants who were non-Hispanic Black, no differences were observed between Hispanic and non-Hispanic White participants in terms of HRQOL and mental health indicators. In part, this may be explained by the complex interplay between cultural and social factors and the healthcare system,^46^ which requires further study. Differences in economic burden were noted between Hispanic and non-Hispanic White participants such that Hispanic participants had significantly more HCP visits, gastroenterologist visits, emergency room visits, hospitalizations, and higher annualized direct medical costs. The increased economic burden evident in Hispanic participants with UC may be due to differences in treatment course and response. For example, a systematic review and meta-analysis found that biologics, a common therapy prescribed in the treatment of moderate/severe UC, were less frequently used among Hispanic patients with UC than among non-Hispanic White patients.^47^ Delayed or lack of initiation of biologics among Hispanic patients and other minority races with UC may be due to concerns about cost, side effects, or physician bias.^48^ In support of this interpretation, in a study of patients receiving biologic treatment, Hispanic patients had higher rates of hospitalization, IBD-related surgery, and infection than non-Hispanic patients, implying that biologics may have a reduced treatment response among the Hispanic group.^48^ As such, Hispanic participants with UC may have a higher economic burden than non-Hispanic White participants due to treatments that may not adequately control their symptoms. Importantly, strategies to acknowledge disparities in treatment responses and economic burden can substantially impact HRQoL and mental health outcomes among different racial/ethnic groups.

The relationship between severity and labor force participation depended on race/ethnicity, such that the odds of labor force participation for Hispanic participants with moderate/severe UC was 3.26 times that of those with mild UC. Notably, among non-Hispanic White participants, roughly equal proportions of participants with mild or moderate/severe UC reported labor force participation. Although our study was unable to evaluate variation in labor types, data from the 2014 US Bureau of Labor Statistics survey showed an overrepresentation of Hispanic individuals in physically demanding industries such as construction, agriculture, and leisure/hospitality.^49^ As such, our results may suggest that the types of employment more commonly held by Hispanic individuals, such as manual labor and skilled trades, are contributing to the observed association between ethnicity and UC severity. This notion is supported by a recent case–control study conducted in Japan, which found that individuals in specific sectors like occupations within service, manufacturing, cleaning, and packing industries were at higher risk of UC.^50^ While a counterargument could be made that those involved in trades or manual labor may be more physically fit than those in sedentary occupations, we hypothesize that intense physical activity may exacerbate UC symptoms; strenuous activity has been inversely associated with mucosal healing and may worsen the pathogenesis and downstream consequences of gastrointestinal diseases like UC.^51,52^

Interestingly, although non-White populations face many of the same societal challenges and barriers to healthcare, the results of this study underscore distinct humanistic and economic challenges among non-Hispanic Black and Hispanic participants with UC compared with their non-Hispanic White counterparts. As characteristics such as education, employment, and household income were controlled for in multivariable analyses, these differences may signify inherent biases and behaviors that are unique to each group. Additionally, the pool of non-Hispanic Black and Hispanic participants surveyed in this study may be heterogenous with respect to ancestry and regional variation and cultural influence. Regardless, studies of PROs among patients of various race/ethnicity groups are complex and may encompass a range of factors that extend beyond the disease itself. Future research is needed to better understand the contribution of the non-Hispanic Black or Hispanic experience to these and other results through a bidirectional lens, as UC severity may not only influence work-related challenges and participation, but work-related challenges may also influence UC severity.

A key strength of this study was the use of PROs, which provide patient perspectives on disease outcomes over third-party perceptions of disease burden. The use of several different PROs to measure an outcome (eg, mental health) allowed us to capture a more holistic picture of health and well-being. Clinical trial study populations have not historically reflected the racially and ethnically diverse national population. As such, increasing racial/ethnic representation in PRO data collection can aid in understanding disease impacts on patient quality of life and help inform clinical care. Furthermore, the use of NHWS data, which is designed to be nationally representative of the US adult population with respect to age, sex, and race/ethnicity, in this study, provides PRO data on a diverse group of patients with UC. The sampling approach used by the NHWS ensures comprehensive capture of racial and ethnic groups, permitting comparisons between various groups. The results of this study, therefore, contribute to race/ethnicity representation in PROs, aiding the understanding of disease impacts on patient quality of life.^53^ The extensive roster of outcomes assessed in this study allows a robust contribution to the literature on a variety of PROs, increases the validity of the study findings through consistency across measures, and contributes to a holistic understanding of patient burden in UC.

Limitations of this study include the self-reported nature of race/ethnicity and disease severity, which could not be independently verified. The relatively small sample sizes of non-Hispanic Black and Hispanic groups in our study, despite pooling data across 3 survey years, may have limited the precision of our estimates. While our study sought to examine racial/ethnic differences in UC-related outcomes among a more diverse sample of respondents, additional categories or an “other races/ethnicities” category lacked sufficient sample for inclusion. Furthermore, the 3 categories used to characterize race/ethnicity may not sufficiently account for the heterogeneity that occurs within racial/ethnic groups. As the survey was conducted in English in a virtual setting, non-English-speaking individuals, elderly groups, and those without reliable access to computer or Internet access could be underrepresented in our sample. The study population was also generally more educated and affluent than historical national averages and may not therefore be reflective of the overall US population. Thus, the generalizability of our findings may be limited to a subset of patients with UC. While there are important generalizability limitations, our findings play a role in bridging the evidence gap. Given the paucity of data, analyzing NHWS data is a step forward and further underscores the significant complexity and nuances. Another limitation is that cost data may underestimate the financial impact in the current post-COVID era, as medical costs were calculated using 2018 data. Additionally, residual confounding may have biased multivariable models, despite attempts to adjust for other potential explanatory variables. Finally, due to the cross-sectional nature of the study, causal inference was not possible.

## Conclusions

Our study demonstrates that patients with moderate/severe UC experience greater humanistic and economic burden than those with mild UC, consistent with previous reports showing a substantial increase in burden with worse disease severity. Furthermore, race/ethnicity played a role in predicting PROs, including mental health, HRQoL, and economic burden. Hispanic participants demonstrated distinct patterns, particularly in how disease severity influenced employment outcomes. Addressing the burden of UC effectively necessitates a nuanced approach that recognizes patient experiences within broader social contexts. Further research is crucial to unravel the factors influencing the interplay between UC severity and race/ethnicity among UC patients, aiming to identify and mitigate healthcare disparities.

## Supplementary Material

otae048_suppl_Supplementary_Table_S1-S4

## Data Availability

Data were obtained from NHWS through a data license agreement and are not publicly available.
