# Supplementary material for: Patient-Reported Racial and Ethnic Disparities in Patients With Ulcerative Colitis: Results From the National Health and Wellness Survey
Source: Crohns Colitis 360. 2024 Sep 18;6(4):otae048. doi: 10.1093/crocol/otae048 (PMC11447937; doi:10.1093/crocol/otae048)
Supplement: otae048_suppl_Supplementary_Table_S1-S4 [file otae048_suppl_supplementary_table_s1-s4.docx]

# Supplemental Materials

Detailed Clinical Characteristics and Study Outcomes

Self-reported UC severity was captured as the participants’ rating of their UC as either mild, moderate, or severe. Participants who used a prescription medication during the study period rated their UC severity while on their medication, whereas participants who did not use prescription medication rated their UC severity in a general sense (i.e., not specific to any symptoms). Patient activation/engagement was assessed using the Patient Activation Measure (PAM), a non-disease-specific measure, where higher scores (scale 0-100) indicate greater levels of engagement.[^1^](#_ENREF_1) The PAM questionnaire includes 13 items with a total score of 0 to 100. Scores can also be grouped into four levels of activation (i.e., Level 1: 0-47, Level 2: 47.1-55.1, Level 3: 55.2-72.4, and Level 4: 72.5-100), where a higher level indicates greater engagement. Previous clinical trial participation data was collected from a non-disease-specific survey question; response options included “yes” or “no”.

Outcomes of interest included PROs of depression and anxiety, HRQoL, labor force participation, WPAI, HCRU, and medical costs. Depression severity was assessed using the Patient Health Questionnaire-9 (PHQ-9), a nine-item validated screening tool for measuring depression symptomology.[^2^](#_ENREF_2) Summed response scores range from 0 to 27. Anxiety severity was assessed using the Generalized Anxiety Disorder Assessment (GAD-7), a seven-item instrument that is used to measure the severity of generalized anxiety disorder (GAD).[^3^](#_ENREF_3) Summed response scores range from 0 to 21 for GAD-7. For both scales, higher scores indicate more severe depression or anxiety.

HRQoL was assessed using two summary scores of the Medical Outcomes Study 36-Item Short Form Survey Instrument (SF-36v2): physical component summary (PCS) and mental component summary (MCS).[^4^](#_ENREF_4) PCS and MCS scores range from 0 to 100, with higher scores indicating better quality of life. The minimum threshold for clinically important difference is 3.0 points.[^5^](#_ENREF_5) The SF-6D and the EuroQol 5-dimension health questionnaire (EQ-5D) instrument were used to assess health state utilities.[^6^](#_ENREF_6) The SF-6D utility scores were generated by applying the SF-36 algorithm to the SF-36v2. The SF-6D index yields summary scores ranging from 0 to 1, where higher scores indicate better quality of life and minimal clinically important difference [MCID] is defined as 0.033 points.[^7^](#_ENREF_7) The EQ-5D instrument consists of the EQ-5D-5L utility index and EQ visual analog scale (VAS). The EQ-5D-5L utility index assesses HRQoL across five dimensions using a five-level rating scale, from no problems to extreme problems. The rating for each item is combined into a 5-digit number, which is mapped to a value set to derive utility values with total scores ranging from -0.224 to 1. This measure has an MCID of 0.028.[^7^](#_ENREF_7) The EQ VAS assesses patient’s self-rated health on a scale from 0 to 100, with the endpoints being ‘worst imaginable health state’ and ‘best imaginable health state’. Higher EQ-5D-5L and EQ VAS scores represent better HRQoL.

Labor force participation was derived from NHWS data through coding employment status as currently in the labor force (i.e., full-time employed, part-time employed, self-employed, or not unemployed but looking for work) or not currently in the labor force (i.e., retired, disabled, homemaker, student, or not employed and not looking for work). Work productivity was assessed using the WPAI questionnaire, a 6-item validated instrument measuring absenteeism (percentage of work time missed because of one's health), presenteeism (percentage of impairment experienced while at work because of one's health), overall work productivity loss (overall impairment estimate), and activity impairment (percentage of impairment in daily activities because of one's health) over the past seven days.[^8^](#_ENREF_8) Only participants who reported a work status of full-time, part-time, or self-employed provided data for absenteeism, presenteeism, and overall work impairment.

HCRU included the number of self-reported visits to any healthcare provider (HCP), gastroenterologists (GE), and emergency rooms (ER), and hospitalizations over the past six months. HCP visits include visits to any of the following: general practitioner/family practitioner, internist, allergist, cardiologist, dentist, dermatologist, diabetologist, endocrinologist, gastroenterologist, geriatrician, gynecologist, hepatologist, infectious disease specialist/infectologist, neurologist, nephrologist, nurse practitioner/physician assistant, obstetrician, oncologist, ophthalmologist, orthopedist, otolaryngologist, plastic surgeon, podiatrist, psychiatrist, psychologist/therapist, pulmonologist, respiratory therapist, rheumatologist, urologist, and other medical specialist. HCRU questions used the phrasing “for your own medical condition” to ensure that trips to accompany a friend or relative for their medical issues were not included in the calculation. The question phrasing was intentionally vague so that HCRU for any medical condition was included. Direct medical costs were imputed using data from the region-specific Medical Expenditure Panel Survey (MEPS) and included costs of an average HCP visit, ER visit, and hospitalization.[^9^](#_ENREF_9) Direct costs were calculated based on the annual number of visits, as estimated by doubling the number of visits self-reported over the past six months, and the average cost for each visit. Indirect costs were those associated with work productivity impairment and were calculated using estimated wages/salaries for each participant with data from the US Bureau of Labor Statistics. Mean absenteeism and presenteeism estimates were each multiplied by hourly wage rates to calculate total lost wages. Annual estimates of indirect costs assumed 50 work weeks in a year. This cost analysis approach has been used in prior research in the NHWS.[^10^](#_ENREF_10)^,^[^11^](#_ENREF_11)

Supplemental Table 1: Multivariable analyses of outcomes by severity among individuals with ulcerative colitis

| **Outcomes** | **UC Severity** | **Adjusted Mean** | **95% CI for Adjusted Mean** | **β or exp(β)** | **95% CI for β** | ***P* value** |
| --- | --- | --- | --- | --- | --- | --- |
| **PHQ-9^a^** | Mild | 6.39 | 6.05 to 6.76 | ref | - | **-** |
|  | Moderate/severe | 8.59 | 7.97 to 9.26 | 1.34 | 1.22 to 1.48 | **<0.001** |
| **GAD-7^a^** | Mild | 4.74 | 4.46 to 5.04 | ref | - | **-** |
|  | Moderate/severe | 6.28 | 5.79 to 6.82 | 1.32 | 1.19 to 1.47 | **<0.001** |
| **MCS^b^** | Mild | 44.06 | 43.43 to 44.70 | ref | - | **-** |
|  | Moderate/severe | 40.19 | 39.30 to 41.07 | -3.88 | -4.99 to -2.77 | **<0.001** |
| **PCS^b^** | Mild | 45.64 | 45.09 to 46.18 | ref | - | **-** |
|  | Moderate/severe | 41.85 | 41.10 to 42.61 | -3.78 | -4.73 to -2.83 | **<0.001** |
| **SF-6D^b^** | Mild | 0.654 | 0.647 to 0.662 | ref | - | **-** |
|  | Moderate/severe | 0.598 | 0.587 to 0.608 | -0.06 | -0.07 to -0.04 | **<0.001** |
| **EQ-5D^b^** | Mild | 0.748 | 0.738 to 0.759 | ref | - | **-** |
|  | Moderate/severe | 0.689 | 0.674 to 0.704 | -0.06 | -0.08 to -0.04 | **<0.001** |
| **EQ VAS^b^** | Mild | 66.79 | 65.23 to 68.36 | ref | - | **-** |
|  | Moderate/severe | 60.21 | 58.03 to 62.40 | -6.58 | -9.31 to -3.84 | **<0.001** |
| **Labor force participation^c^** | Mild | 54.9% | 51.1% to 58.8% | ref | - | **-** |
|  | Moderate/severe | 60.1% | 54.6% to 65.3% | 1.23 | 0.94 to 1.63 | 0.135 |
| **Absenteeism^a^** | Mild | 13.38 | 10.66 to 16.80 | ref | - | **-** |
|  | Moderate/severe | 21.82 | 16.15 to 29.47 | 1.63 | 1.16 to 2.30 | **0.005** |
| **Presenteeism^a^** | Mild | 29.27 | 26.57 to 32.25 | ref | - | **-** |
|  | Moderate/severe | 45.67 | 40.46 to 51.56 | 1.56 | 1.36 to 1.79 | **<0.001** |
| **Overall work productivity impairment^a^** | Mild  Moderate/severe | 32.53 | 29.48 to 35.89 | ref | - | **-** |
|  |  | 52.14 | 46.08 to 59.01 | 1.60 | 1.39 to 1.85 | **<0.001** |
| **Activity impairment^a^** | Mild | 33.82 | 32.11 to 35.61 | ref | - | **-** |
|  | Moderate/severe | 48.63 | 45.27 to 52.25 | 1.44 | 1.32 to 1.57 | **<0.001** |
| **HCP visits^a^** | Mild | 5.85 | 5.45 to 6.29 | ref | - | **-** |
|  | Moderate/severe | 6.77 | 6.13 to 7.47 | 1.16 | 1.02 to 1.31 | **0.021** |
| **GE visits^a^** | Mild | 0.45 | 0.39 to 0.52 | ref | - | **-** |
|  | Moderate/severe | 0.73 | 0.61 to 0.87 | 1.61 | 1.29 to 2.03 | **<0.001** |
| **ER visits^a^** | Mild | 0.40 | 0.34 to 0.46 | ref | - | **-** |
|  | Moderate/severe | 0.66 | 0.55 to 0.80 | 1.68 | 1.32 to 2.13 | **<0.001** |
| **Hospitalizations^a^** | Mild | 0.31 | 0.25 to 0.40 | ref | - | **-** |
|  | Moderate/severe | 0.46 | 0.34 to 0.62 | 1.47 | 1.01 to 2.13 | **0.042** |
| **Annualized direct medical costs^a^** | Mild | $39,200.33 | $34,954.42 to $43,961.99 | ref | - | **-** |
|  | Moderate/severe | $50,252.95 | $42,775.34 to $59,037.74 | 1.28 | 1.05 to 1.57 | **0.016** |
| **Annualized indirect costs^a^** | Mild | $11,856.60 | $10,498.08 to $13,390.93 | ref | - | **-** |
|  | Moderate/severe | $16,181.29 | $13,810.87 to $18,958.56 | 1.36 | 1.14 to 1.64 | **0.001** |

^a^ Modeled using a log link with a negative binomial distribution; exp(β) = rate ratio.

^b^ Modeled using an identity link with a normal distribution; β = the estimated mean difference.

^c^ Modeled using a logit link with a binomial distribution; exp(β) = odds ratio.

Note: Models control for age (continuous; set to mean = 45.19 years), gender (male [ref]; female), marital status (single/never married/decline to answer; married/living with a partner [ref]), educational attainment (less than a college degree/declined to answer; college graduate or higher [ref]), household income (<$25,000; $25,000 to <$50,000; $50,000 to <$100,000; $100,00+ [ref]), health insurance coverage (Medicare; Medicaid/VA/CHAMPUS; uninsured; commercial/TRICARE/don't know [ref]), weight status (obese; overweight; underweight/normal weight/ declined to answer [ref]), smoking status (current smoker; former smoker; never smoker [ref]), alcohol use (drinks alcohol; does not drink alcohol [ref]), and Charlson Comorbidity Index Score (continuous; set to mean = 1.14)

Abbreviations: EQ-5D-5L = EuroQol 5-Dimension 5-Level; ER = emergency room; GAD-7 = Generalized Anxiety Disorder – 7 item; GE = gastroenterologist; HCP = healthcare provider; HCRU = healthcare resource utilization; MCS = Mental Component Summary; PCS = Physical Component Summary; PHQ-9 = Patient Health Questionnaire – 9 item; SD = standard deviation; SF-6D = Short Form – 6 Dimension; WPAI = Work Productivity and Activity Impairment; VAS = Visual Analog Scale.

**Supplemental Table 2: Bivariate analyses of outcomes by severity among individuals with ulcerative colitis**

|  | **Mild**  **(n = 1,070)** | | | **Moderate/severe**  **(n = 557)** | | | ***P* value** |
| --- | --- | --- | --- | --- | --- | --- | --- |
|  | **Valid n** | **Mean (SD)** | **Median (min, max)** | **Valid n** | **Mean (SD)** | **Median (min, max)** |  |
| **HRQoL**  PHQ-9^a^  GAD-7^b^  MCS^c^  PCS^c^  SF-6D^c^  EQ-5D  EQ VAS | 984  984  984  984  984  984  984 | 6.78 (6.71)  5.08 (5.32)  45.08 (11.74)  46.01 (9.94)  0.666 (0.141)  0.760 (0.174)  67.63 (25.30) | 4.00 (0.00, 27.00)  3.00 (0.00, 21.00)  46.60 (10.58, 71.00)  47.41 (17.78, 68.38)  0.650 (0.320, 1.00)  0.803 (-0.109, 1.00)  75.00 (1.00, 100.00) | 516  516  516  516  516  516  516 | 10.62 (7.26)  8.03 (5.71)  38.25 (11.01)  41.15 (9.29)  0.575 (0.123)  0.666 (0.200)  58.62 (27.15) | 10.00 (0.00, 27.00)  8.00 (0.00, 21.00)  36.21 (7.53, 65.71)  41.12 (16.88, 66.23)  0.560 (0.300, 0.960)  0.710 (-0.109, 100.00)  65.00 (0.00, 100.00) | **<0.001**  **<0.001**  **<0.001**  **<0.001**  **<0.001**  **<0.001**  **<0.001** |
| **Labor force participation, n (%)** | 984 | 523 (53.2%) | - | 516 | 311 (60.3%) | - | **0.008** |
| **WPAI**  Absenteeism^d^  Presenteeism^d^  Overall work productivity impairment  Activity impairment | 496  493  486  984 | 14.52 (25.08)  31.12 (32.34)  34.48 (35.19)  35.44 (31.05) | 0.00 (0.00,100.00)  20.00 (0.00,100.00)  20.00 (0.00,100.00)  30.00 (0.00,100.00) | 300  304  296  516 | 28.18 (27.24)  52.80 (30.01)  60.94 (32.44)  54.58 (29.01) | 22.50 (0.00,100.00)  60.00 (0.00,100.00)  72.00 (0.00,100.00)  60.00 (0.00,100.00) | **<0.001**  **<0.001**  **<0.001**  **<0.001** |
| **HCRU**  HCP visits^e^  GE visits  ER visits  Hospitalizations | 984  984  984  984 | 6.20 (8.40)  0.50 (1.02)  0.62 (2.05)  0.68 (3.96) | 4.00 (0.00, 132.00)  0.00 (0.00, 12.00)  0.00 (0.00, 35.00)  0.00 (0.00, 74.00) | 516  516  516  516 | 8.33 (21.62)  0.87 (3.33)  1.51 (4.05)  1.16 (3.17) | 5.00 (0.00, 440.00)  0.00 (0.00, 56.00)  0.00 (0.00, 60.00)  0.00 (0.00, 45.00) | **0.006**  **<0.001**  **<0.001**  **0.019** |
| **Costs**  Annualized direct medical costs  Annual indirect costs^f^ | 984  516 | $48,268.04 ($166,529.65)  $12,225.00 ($16,871.20) | $17,108.00  ($0.00, $3,781,664.00)  $5,459.06  ($0.00, $176,400.00) | 516  303 | $70,242.68 ($142,928.12)  $19,167.47 ($19,190.74) | $29,526.00  (0.00, $1,699,322.00)  $13,608.75  (0.00, $107,572.50) | **0.011**  **<0.001** |

^a^ PHQ-9 includes nine items (range of 0 to 27) where higher scores indicate mores severe depression.

^b^ GAD-7 includes seven items (range 0 to 21) and a higher score indicates more severe general anxiety disorder.
^c^ Differences in 3 points on the norm-based component summary scores and 0.041 points on health utilities represent clinically meaningful differences.
^d^ Absenteeism was not calculated for those who worked zero hours and missed zero hours in the last seven days and presenteeism was only asked among those who worked more than zero hours in the last seven days.

^e^ Includes visits to any of the following: general practitioner/family practitioner; internist; allergist; cardiologist; dentist; dermatologist; diabetologist; endocrinologist; gastroenterologist; geriatrician; gynecologist; hepatologist; infectious disease specialist/infectologist (diseases such as HIV or hepatitis); neurologist; nephrologist; nurse practitioner/physician assistant; obstetrician; oncologist; ophthalmologist; orthopedist; otolaryngologist (ears, nose, and throat specialist); plastic surgeon; podiatrist; psychiatrist; psychologist/therapist; pulmonologist (lung specialist); respiratory therapist; rheumatologist; urologist; other medical specialist.

^f^ Total annual indirect costs were only calculated among respondents who were participating in the labor force at the time of the survey and who had a valid response (i.e., non-missing) for the number of hours working in the past 7 days and the number of hours missed in the past 7 days.

Note: Comparisons that reached statistical significance and highlighted in bold text. Chi-square tests were used to determine significant differences for categorical variables. ANOVA and independent sample *t* tests were used for continuous variables. *P* values (α=0.05) were provided for the omnibus test and pairwise testing between race/ethnicity subgroups. Statistical significance was adjusted for multiplicity using the Bonferroni correction for multiple comparisons. Results from bivariate analyses were used to identify covariates for the multivariable models (variables that were associated with the key independent variables or were of theoretical importance).
Abbreviations: EQ-5D-5L = EuroQol 5-Dimension 5-Level; ER = emergency room; GAD-7 = Generalized Anxiety Disorder – 7 item; GE = gastroenterologist; HCP = healthcare provider; HCRU = healthcare resource utilization; MCS = Mental Component Summary; PCS = Physical Component Summary; PHQ-9 = Patient Health Questionnaire – 9 item; SD = standard deviation; SF-6D = Short Form – 6 Dimension; WPAI = Work Productivity and Activity Impairment; VAS = Visual Analog Scale.

**Supplemental Table 3: Multivariable analyses of outcomes by race/ethnicity among individuals with ulcerative colitis**

| **Outcomes** | **Race/Ethnicity** | **Adjusted Mean** | **95% CI for Adjusted Mean** | **β or exp(β)** | **95% CI for β** | ***P* value** |
| --- | --- | --- | --- | --- | --- | --- |
| **PHQ-9^a^** | Non-Hispanic White | 7.16 | 6.81 to 7.54 | - | - | - |
|  | Non-Hispanic Black | **5.88** | **4.92 to 7.02** | **0.82** | **0.68 to 0.99** | **0.038** |
|  | Hispanic | 7.20 | 6.44 to 8.04 | 1.00 | 0.89 to 1.14 | 0.946 |
| **GAD-7^a^** | Non-Hispanic White | 5.25 | 4.96 to 5.56 | - | - | - |
|  | Non-Hispanic Black | **4.25** | **3.50 to 5.17** | **0.81** | **0.66 to 0.99** | **0.044** |
|  | Hispanic | 5.54 | 4.91 to 6.25 | 1.05 | 0.92 to 1.21 | 0.439 |
| **MCS^b^** | Non-Hispanic White | 42.52 | 41.93 to 43.11 | - | - | - |
|  | Non-Hispanic Black | **45.42** | **43.38 to 47.47** | **2.91** | **0.75 to 5.06** | **0.008** |
|  | Hispanic | 42.63 | 41.33 to 43.93 | 0.11 | -1.34 to 1.57 | 0.879 |
| **PCS^b^** | Non-Hispanic White | 44.26 | 43.75 to 44.77 | - | - | - |
|  | Non-Hispanic Black | 45.28 | 43.53 to 47.03 | 1.02 | -0.82 to 2.86 | 0.278 |
|  | Hispanic | 44.31 | 43.20 to 45.42 | 0.05 | -1.20 to 1.30 | 0.938 |
|  | Non-Hispanic White | 0.635 | 0.628 to 0.642 | - | - | - |
| **SF-6D^b^** | Non-Hispanic Black | 0.652 | 0.629 to 0.676 | 0.02 | -0.01 to 0.04 | 0.169 |
|  | Hispanic | 0.626 | 0.611 to 0.641 | -0.01 | -0.03 to 0.01 | 0.283 |
|  | Non-Hispanic White | 0.729 | 0.719 to 0.738 | - | - | - |
| **EQ-5D^b^** | Non-Hispanic Black | **0.775** | **0.741 to 0.809** | **0.05** | **0.01 to 0.08** | **0.010** |
|  | Hispanic | 0.705 | 0.684 to 0.727 | -0.02 | -0.05 to 0.00 | 0.055 |
|  | Non-Hispanic White | 63.76 | 62.30 to 65.22 | - | - | - |
| **EQ VAS^b^** | Non-Hispanic Black | **70.60** | **65.56 to 75.65** | **6.84** | **1.52 to 12.16** | **0.012** |
|  | Hispanic | 65.65 | 62.45 to 68.85 | 1.89 | -1.71 to 5.48 | 0.304 |
| **Labor force participation^c^** | Non-Hispanic White | 55.2% | 51.5% to 58.7% | - | - | - |
|  | Non-Hispanic Black | 66.9% | 53.9% to 77.8% | 1.65 | 0.93 to 2.92 | 0.089 |
|  | Hispanic | 59.7% | 51.6% to 67.2% | 1.20 | 0.84 to 1.73 | 0.321 |
|  | Non-Hispanic White | 15.64 | 12.38 to 19.76 | - | - | - |
| **Absenteeism^a^** | Non-Hispanic Black | 12.84 | 7.30 to 22.58 | 0.82 | 0.44 to 1.54 | 0.537 |
|  | Hispanic | 18.19 | 12.55 to 26.37 | 1.16 | 0.76 to 1.77 | 0.483 |
|  | Non-Hispanic White | 33.87 | 30.79 to 37.26 | - | - | - |
| **Presenteeism^a^** | Non-Hispanic Black | 30.71 | 24.16 to 39.04 | 0.91 | 0.70 to 1.17 | 0.454 |
|  | Hispanic | 36.75 | 31.37 to 43.06 | 1.09 | 0.91 to 1.30 | 0.367 |
| **Overall work productivity impairment^a^** | Non-Hispanic White | 37.91 | 34.40 to 41.78 | - | - | - |
|  | Non-Hispanic Black | 33.61 | 26.40 to 42.78 | 0.89 | 0.68 to 1.15 | 0.362 |
|  | Hispanic | 42.00 | 35.68 to 49.43 | 1.11 | 0.92 to 1.33 | 0.271 |
| **Activity impairment^a^** | Non-Hispanic White | 38.21 | 36.40 to 40.10 | - | - | - |
|  | Non-Hispanic Black | 36.98 | 31.25 to 43.76 | 0.97 | 0.81 to 1.16 | 0.719 |
|  | Hispanic | 39.40 | 35.45 to 43.79 | 1.03 | 0.92 to 1.16 | 0.612 |
|  | Non-Hispanic White | 5.77 | 5.39 to 6.16 | - | - | - |
| **HCP visits^a^** | Non-Hispanic Black | 6.80 | 5.39 to 8.59 | 1.18 | 0.92 to 1.51 | 0.189 |
|  | Hispanic | **7.96** | **6.90 to 9.18** | **1.38** | **1.17 to 1.62** | **<0.001** |
|  | Non-Hispanic White | 0.49 | 0.43 to 0.56 | - | - | - |
| **GE visits^a^** | Non-Hispanic Black | 0.39 | 0.24 to 0.62 | 0.78 | 0.48 to 1.27 | 0.319 |
|  | Hispanic | **0.84** | **0.65 to 1.08** | **1.70** | **1.26 to 2.28** | **<0.001** |
|  | Non-Hispanic White | 0.43 | 0.37 to 0.49 | - | - | - |
| **ER visits^a^** | Non-Hispanic Black | 0.55 | 0.36 to 0.84 | 1.30 | 0.84 to 2.02 | 0.244 |
|  | Hispanic | **0.71** | **0.55 to 0.92** | **1.68** | **1.26 to 2.23** | **<0.001** |
|  | Non-Hispanic White | 0.31 | 0.25 to 0.39 | - | - | - |
| **Hospitalizations^a^** | Non-Hispanic Black | 0.51 | 0.27 to 0.97 | 1.66 | 0.85 to 3.23 | 0.136 |
|  | Hispanic | **0.60** | **0.40 to 0.89** | **1.94** | **1.25 to 3.03** | **0.003** |
| **Annualized direct medical costs^a^** | Non-Hispanic White | $38,240.01 | $34,366.77 to $42,549.78 | - | - | - |
|  | Non-Hispanic Black | $56,515.66 | $38,842.61 to $82,229.79 | 1.48 | 1.00 to 2.19 | 0.053 |
|  | Hispanic | **$63,348.65** | **$49,977.83 to $80,296.63** | **1.66** | **1.27 to 2.16** | **<0.001** |
| **Annualized indirect costs^a^** | Non-Hispanic White | $13,603.51 | $12,054.41 to $15,351.69 | - | - | - |
|  | Non-Hispanic Black | **$8,830.11** | **$6,498.21 to $11,998.83** | **0.65** | **0.47 to 0.90** | **0.010** |
|  | Hispanic | $13,446.17 | $10,987.29 to $16,455.34 | 0.99 | 0.79 to 1.24 | 0.920 |

^a^ Modeled using a log link with a negative binomial distribution; exp(β) = rate ratio.

^b^ Modeled using an identity link with a normal distribution; β = the estimated mean difference

^c^ Modeled using a logit link with a binomial distribution; exp(β) = odds ratio

Note: Models control for age (continuous; set to mean = 45.19 years), gender (male [ref]; female), marital status (single/never married/decline to answer; married/living with a partner [ref]), educational attainment (less than a college degree/declined to answer; college graduate or higher [ref]), household income (<$25,000; $25,000 to <$50,000; $50,000 to <$100,000; $100,00+ [ref]), health insurance coverage (Medicare; Medicaid/VA/CHAMPUS; uninsured; commercial/TRICARE/don't know [ref]), weight status (obese; overweight; underweight/normal weight/ declined to answer [ref]), smoking status (current smoker; former smoker; never smoker [ref]), alcohol use (drinks alcohol; does not drink alcohol [ref]), and Charlson Comorbidity Index Score (continuous; set to mean = 1.14)

Abbreviations: EQ-5D-5L = EuroQol 5-Dimension 5-Level; ER = emergency room; GAD-7 = Generalized Anxiety Disorder – 7 item; GE = gastroenterologist; HCP = healthcare provider; HCRU = healthcare resource utilization; MCS = Mental Component Summary; PCS = Physical Component Summary; PHQ-9 = Patient Health Questionnaire – 9 item; SD = standard deviation; SF-6D = Short Form – 6 Dimension; WPAI = Work Productivity and Activity Impairment; VAS = Visual Analog Scale.

**Supplemental Table 4: Bivariate analyses of outcomes by race/ethnicity among individuals with ulcerative colitis**

|  | **Non-Hispanic White**  **(n = 818)** | **Non-Hispanic Black**  **(n = 109)** | **Hispanic**  **(n = 150)** | ***P* value** | | | |
| --- | --- | --- | --- | --- | --- | --- | --- |
|  |  |  |  | **Omnibus** | **Bonferroni-adjusted pairwise comparisons** | | |
|  |  |  |  |  | **Non-Hispanic White vs. Non-Hispanic Black** | **Non-Hispanic White vs. Hispanic** | **Non-Hispanic Black vs. Hispanic** |
| **HRQoL, mean (SD)**  PHQ-9^a^  GAD-7^b^  MCS^c^  PCS^c^  SF-6D^c^  EQ-5D  EQ VAS | 7.51 (7.02)  5.57 (5.59)  43.67 (12.24)  44.46 (10.33)  0.647 (0.141)  0.739 (0.185)  64.43 (25.91) | 8.57 (6.70)  6.53 (5.21)  41.96 (11.02)  44.61 (8.48)  0.618 (0.156)  0.747 (0.159)  67.75 (28.53) | 10.65 (7.28)  8.31 (5.47)  38.71 (9.93)  43.67 (8.91)  0.586 (0.126)  0.670 (0.206)  63.73 (27.16) | **<0.001**  **<0.001**  **<0.001**  0.508  **<0.001**  **<0.001**  0.421 | 0.454  0.305  0.497  >0.99  0.145  >0.99  0.685 | **<0.001**  **<0.001**  **<0.001**  0.776  **<0.001**  **<0.001**  >0.99 | **0.038**  **0.021**  0.062  >0.99  0.165  **0.002**  0.594 |
| **Labor force participation, n (%)** | 587 (51.0%) | 70 (70.7%) | 177 (70.5%) | **<0.001** | **0.001** | **<0.001** | >0.99 |
| **WPAI, mean % (SD)**  Absenteeism^d^  Presenteeism^d^  Overall work productivity impairment  Activity impairment | 16.26 (25.68)  35.46 (33.05)  39.23 (35.94)  40.28 (31.63) | 24.43 (28.78)  42.06 (34.01)  50.24 (38.80)  45.45 (35.72) | 29.51 (26.84)  51.30 (30.35)  60.28 (32.45)  48.45 (29.25) | **<0.001**  **<0.001**  **<0.001**  **<0.001** | **0.046**  0.346  **0.049**  0.352 | **<0.001**  **<0.001**  **<0.001**  **<0.001** | 0.538  0.146  0.153  >0.99 |
| **HCRU, mean (SD)**  HCP visits^e^  GE visits  ER visits  Hospitalizations | 6.11 (6.70)  0.54 (1.00)  0.66 (2.56)  0.60 (3.22) | 7.32 (8.98)  0.41 (0.83)  1.44 (2.39)  1.40 (2.32) | 10.55 (31.50)  1.10 (4.68)  1.93 (4.20)  1.76 (5.63) | **<0.001**  **<0.001**  **<0.001**  **<0.001** | >0.99  >0.99  **0.030**  0.112 | **<0.001**  **0.001**  **<0.001**  **<0.001** | 0.175  **0.019**  0.475  >0.99 |
| **Costs, mean (SD)**  Annualized direct medical costs  Annual indirect costs^f^ | $45,720.64  (147,648.88)  $14,344.01  ($18,673.10) | $74,117.01  ($90,923.77)  $12,652.20  ($16,338.89) | $94,918.93  ($214,960.76)  $17,240.01  ($16,398.87) | **<0.001**  0.1094 | 0.260  >0.99 | **<0.001**  0.202 | 0.803  0.223 |

^a^ PHQ-9 includes nine items (range of 0 to 27) where higher scores indicate mores severe depression.

^b^ GAD-7 includes seven items (range 0 to 21) and a higher score indicates more severe general anxiety disorder.
^c^ Differences in 3 points on the norm-based component summary scores and 0.041 points on health utilities represent clinically meaningful differences.
^d^ Absenteeism was not calculated for those who worked zero hours and missed zero hours in the last seven days and presenteeism was only asked among those who worked more than zero hours in the last seven days.

^e^ Includes visits to any of the following: general practitioner/family practitioner; internist; allergist; cardiologist; dentist; dermatologist; diabetologist; endocrinologist; gastroenterologist; geriatrician; gynecologist; hepatologist; infectious disease specialist/infectologist (diseases such as HIV or hepatitis); neurologist; nephrologist; nurse practitioner/physician assistant; obstetrician; oncologist; ophthalmologist; orthopedist; otolaryngologist (ears, nose, and throat specialist); plastic surgeon; podiatrist; psychiatrist; psychologist/therapist; pulmonologist (lung specialist); respiratory therapist; rheumatologist; urologist; other medical specialist.

^f^ Total annual indirect costs were only calculated among respondents who were participating in the labor force at the time of the survey and who had a valid response (i.e., Non-missing) for the number of hours working in the past 7 days and the number of hours missed in the past 7 days.

Note: Comparisons that reached statistical significance and highlighted in bold text. Chi-square tests were used to determine significant differences for categorical variables. ANOVA and independent sample *t* tests were used for continuous variables. *P* values (α=0.05) were provided for the omnibus test and pairwise testing between race/ethnicity subgroups. Statistical significance was adjusted for multiplicity using the Bonferroni correction for multiple comparisons. Results from bivariate analyses were used to identify covariates for the multivariable models (variables that were associated with the key independent variables or were of theoretical importance).
Abbreviations: EQ-5D-5L = EuroQol 5-Dimension 5-Level; ER = emergency room; GAD-7 = Generalized Anxiety Disorder – 7 item; GE = gastroenterologist; HCP = healthcare provider; HCRU = healthcare resource utilization; MCS = Mental Component Summary; PCS = Physical Component Summary; PHQ-9 = Patient Health Questionnaire – 9 item; SD = standard deviation; SF-6D = Short Form – 6 Dimension; WPAI = Work Productivity and Activity Impairment; VAS = Visual Analog Scale.

# References

1. Hibbard JH, Stockard J, Mahoney ER, Tusler M. Development of the Patient Activation Measure (PAM): conceptualizing and measuring activation in patients and consumers. *Health Serv Res*. Aug 2004;39(4 Pt 1):1005-26. doi:10.1111/j.1475-6773.2004.00269.x

2. Kroenke K, Spitzer RL, Williams JB. The PHQ-9: validity of a brief depression severity measure. *J Gen Intern Med*. Sep 2001;16(9):606-13. doi:10.1046/j.1525-1497.2001.016009606.x

3. Spitzer RL, Kroenke K, Williams JB, Löwe B. A brief measure for assessing generalized anxiety disorder: the GAD-7. *Arch Intern Med*. May 22 2006;166(10):1092-7. doi:10.1001/archinte.166.10.1092

4. Ware J, Kosinski M, Keller S. SF-36 physical and mental health summary scales. *A user's manual*. 2001;1994

5. Ware J, Snow K, Kosinski M, Gandek B. *SF-36 Health Survey Manual and Interpretation Guide*. Nimrod Press; 1992.

6. Herdman M, Gudex C, Lloyd A, et al. Development and preliminary testing of the new five-level version of EQ-5D (EQ-5D-5L). *Qual Life Res*. Dec 2011;20(10):1727-36. doi:10.1007/s11136-011-9903-x

7. Walters SJ, Brazier JE. Comparison of the minimally important difference for two health state utility measures: EQ-5D and SF-6D. *Qual Life Res*. Aug 2005;14(6):1523-32. doi:10.1007/s11136-004-7713-0

8. Reilly MC, Zbrozek AS, Dukes EM. The validity and reproducibility of a work productivity and activity impairment instrument. *Pharmacoeconomics*. Nov 1993;4(5):353-65. doi:10.2165/00019053-199304050-00006

9. Agency for Healthcare Research and Quality. Medical Expenditure Panel Survey. Available at: <https://www.meps.ahrq.gov/mepstrends/hc_use/>. Accessed November 18, 2021.

10. Dibonaventura M, Gupta S, McDonald M, Sadosky A. Evaluating the health and economic impact of osteoarthritis pain in the workforce: results from the National Health and Wellness Survey. *BMC Musculoskelet Disord*. Apr 28 2011;12:83. doi:10.1186/1471-2474-12-83

11. Dibonaventura MD, Gupta S, McDonald M, Sadosky A, Pettitt D, Silverman S. Impact of self-rated osteoarthritis severity in an employed population: cross-sectional analysis of data from the national health and wellness survey. *Health Qual Life Outcomes*. Mar 15 2012;10:30. doi:10.1186/1477-7525-10-30
